# Facilitating green ammonia manufacture under milder conditions: what do heterogeneous catalyst formulations have to offer?

**DOI:** 10.1039/d1sc04734e

**Published:** 2021-12-01

**Authors:** Manoj Ravi, Joshua W. Makepeace

**Affiliations:** School of Chemistry, University of Birmingham Birmingham B15 2TT UK m.ravi@bham.ac.ukJoshua j.w.makepeace@bham.ac.uk

## Abstract

Ammonia production is one of the largest industrial processes, and is currently responsible for over 1.5% of global greenhouse gas emissions. Decarbonising this process, yielding ‘green ammonia’, is critical not only for sustainable fertilizer production, but also to unlocking ammonia's potential as a zero-carbon fuel and hydrogen store. In this perspective, we critically assess the role of cutting-edge heterogeneous catalysts to facilitate milder ammonia synthesis conditions that will help unlock cheaper, smaller-scale, renewables-coupled ammonia production. The highly-optimised performance of catalysts under the high temperatures and pressures of the Haber–Bosch process stands in contrast to the largely mediocre activity levels reported at lower temperatures and pressures. We identify the recent advances in catalyst design that help overcome the sluggish kinetics of nitrogen activation under these conditions and undertake a categorized analysis of improved activity achieved in a range of heterogeneous catalysts. Building on these observations, we develop a ‘catalyst efficiency’ analysis which helps uncover the success of a holistic approach — one that addresses the issues of nitrogen activation, hydrogenation of adsorbed nitrogen species, and engineering of materials to maximize the utilization of active sites — for achieving the elusive combination of high-activity, low-temperature formulations. Furthermore, we present a discussion on the industrial considerations to catalyst development, emphasising the importance of catalyst lifetime in addition to catalyst activity. This assessment is critical to ensuring that high productivities can translate into real advances in commercial ammonia synthesis.

## Abstract 2

The transition to green ammonia is necessary, but conventional catalysts perform poorly under ‘green’ reaction conditions. We provide a perspective on the recent progress in developing more efficient catalysts for lower temperatures and pressures.

## Graphical abstract

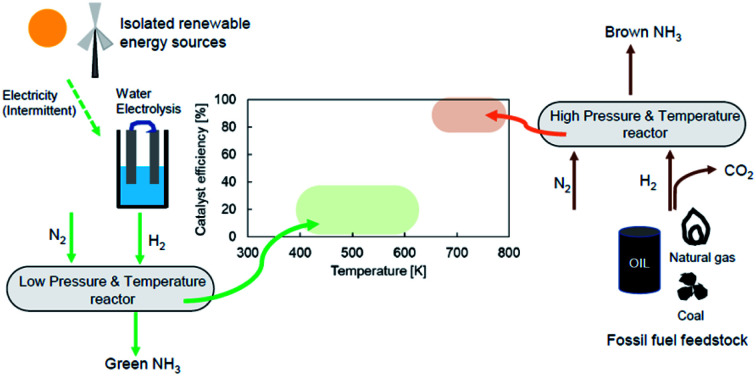

## Introduction

From playing a pivotal role in feeding the world population since the turn of the 20^th^ century, to burgeoning interests in its potential use as a carbon-free fuel and energy store, ammonia's contribution in supporting human life is immense.^1^ That ammonia has emerged as the common denominator in confronting two distinct global challenges of the preceding and the current century — food security and sustainable energy, respectively — is down to its versatility to serve as a vector for both nitrogen and hydrogen.

Ammonia-based fertilizers serve as a source of nitrogen for agricultural crops. The industrial manufacture of fertilizers has enabled a significant increase in agricultural productivity, so much so that nearly half the crops grown worldwide today rely on their use.^1,2^ Looking ahead to the global endeavor to transition to a sustainable energy-based society, the issue of intermittency associated with most renewable energy sources, as well as the need for alternative transportation, mandates the deployment of efficient energy storage and utilization technologies. Among the many energy carriers that are explored for this purpose, hydrogen is one of the leading candidates.^3^ The several routes for its production from renewable energy sources (thermo- and electro-chemical), as well as for its utilization (fuel cells and combustion) contribute to the long-standing interest in a ‘hydrogen economy’.

**Fig. 1:**
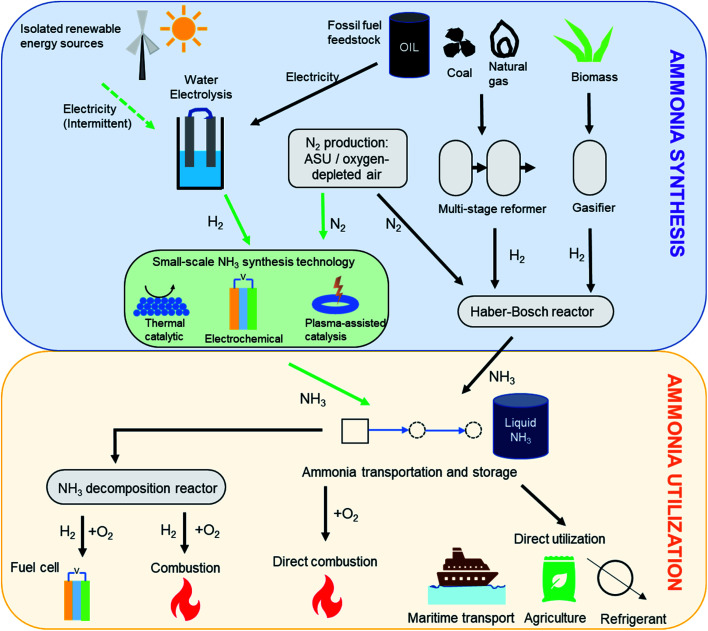
Ammonia synthesis and utilization pathways. Pathway for ‘green ammonia’ synthesis is shown using green arrows.

While hydrogen is characterized by an impressive gravimetric energy density (33 kW h kg^−1^) that compares favorably to that of gasoline, its low volumetric density (3 W h L^−1^ under ambient conditions) presents difficulties for storage.^4^ Liquefaction or compression can help improve the volumetric density, but this requires very low temperatures (−253 °C) and very high pressures (400–700 bar), respectively. It is in this context of efficient hydrogen storage technologies that ammonia is envisaged to play a key role.^5^ Besides its high gravimetric (17.8 wt%) and volumetric (121 kg m^−3^) hydrogen densities,^2^ ammonia liquefies at modest pressures (10 bar) or with mild refrigeration (−33 °C), making it much more feasible for long-distance transport and long-duration storage. Furthermore, being an already widely manufactured industrial chemical, a robust global distribution infrastructure for megatonnes of ammonia each year is already in place. Therefore, the ease of storage and transportation associated with ammonia along with its high energy density (3 kW h kg^−1^) add to its appeal as a hydrogen store and a low-carbon fuel.^5,6^ Importantly, ammonia also offers flexibility in its end-use in the energy and transportation sectors, as detailed in Fig. 1. Considered as a hydrogen store, it can be catalytically cracked to yield hydrogen, which can in turn cater to a number of applications. However, it also can be used directly as a fuel (alone or as a mixed fuel) in combustion or high temperature fuel cells. This ease of storage and flexible end-use have prompted commercial consideration of ammonia as a vector for the export of cheap renewable electricity to locations with less favorable wind and solar conditions, with a number of large-scale projects currently under development.^7,8^

Consideration of ammonia's potential use as a zero-carbon fuel cannot be done in isolation from other intersecting environmental concerns,^9^ chief among which is the impact of synthetic nitrogen fixation on the global nitrogen cycle.^10^ Excess active nitrogen unbalances ecological systems, and ammonia emissions are connected with the formation of particulate air pollution,^11^ highlighting the need for controls on ammonia release into the wider environment. However, it is worth noting that unlike agricultural uses, the purpose of energy-related ammonia use would not be to release active nitrogen into the environment; indeed, the purpose is to convert back to dinitrogen and water, and utilize the energy released in that process. As such, use of ammonia in the context of energy storage should have far less implication for the global nitrogen cycle. However, the direct combustion of ammonia can result in NO_*x*_ gas formation. Mitigation measures such as the selection of appropriate fuel to air ratios (as NH_3_ is used to remove NO_*x*_) are under investigation^12,13^ but may also require appropriate emission control measures.

Ammonia's prominence in shaping modern human history was enabled by the efforts of Fritz Haber and Carl Bosch, who pioneered the industrial ammonia manufacturing process more than a century ago, presenting a much more energy-efficient alternative to the Frank–Caro nitrogen fixation reaction.^14^ The Haber–Bosch (HB) process for ammonia synthesis from molecular nitrogen and hydrogen accounts for 96% of the global annual production of 176 million metric tonnes.^15^ The conventional hydrogen supply for ammonia synthesis is fossil fuel-based (Fig. 1), with natural gas being the primary feedstock because of its abundance and low cost.^15^ Considering the stoichiometry and exothermicity of ammonia synthesis (eqn (1)), the reaction is thermodynamically favored at higher pressure and lower temperature, in accordance with Le Chatelier's principle. However, from the standpoint of kinetics, a lower reaction temperature poses challenges for nitrogen activation and results in a slower rate of reaction. Hence, industrial Haber–Bosch synthesis is typically performed at high temperature (723–873 K) and elevated pressure (150–400 bar). Under these conditions, a 3 : 1 mixture of hydrogen and nitrogen is reacted over an iron-based catalyst to yield a single-pass conversion in the range of 15–25%.^16^ The recycling of unreacted gases allows the overall conversion to approach 97%.^17^ The separation of ammonia from unreacted nitrogen and hydrogen is typically achieved by condensation.

1 N_2_(g) + 3H_2_(g) ↔ 2NH_3_(g) Δ*H*_298K_ = −46 kJ mol^−1^

The high temperatures and pressures associated with HB synthesis result in the process being energy-intensive (30 GJ per tonne-NH_3_ compared with the lower heating value for ammonia of 18.6 GJ per tonne).^18^ Besides the high operational costs that these reaction conditions incur, the use of high-pressure compressors and reactor in the synthesis loop result in significant capital costs. For such processes, the capital cost has been found to increase with plant capacity raised to the power *n* = 0.6–0.8 (eqn (2)); meaning a doubling of the capacity results in a 12–25% decrease in investment per tonne of product.^19,20^ Specifically, for a small-scale Haber–Bosch process, a power factor of *n* = 0.67 has been prescribed for the capital cost correlation.^21,22^

2 Total capital cost = *A*(capacity)^*n*^

This ‘economy of scale’ benefit is the main reason for the centralized nature of ammonia production worldwide, with plant production capacities generally exceeding 1000 tonnes per day.^23^ The economy of scale, as applied above to the ammonia synthesis loop, can also be extended to the on-site production of hydrogen from natural gas. The steam reforming of natural gas to yield hydrogen also requires extreme reaction conditions (1073–1273 K) and is characterized by modest thermal efficiencies (*ca.* 65%).^24,25^ The power law for capital cost applies not only for plant capacity but also for energy loss.^19^ Consequently, reforming processes with mediocre thermal efficiencies are only economical on a large scale.^25,26^ With regards to nitrogen supply for ammonia synthesis, while hollow fibre-based membranes enable efficient air separation on all scales,^27^ conventional air separation units (ASU) that provide nitrogen for many ammonia synthesis plants have also been found to benefit from economies of scale.^17,20^ Therefore, the conventional ammonia manufacturing process as a whole is ideally suited for large-scale operation. Despite attempts being made to adapt the Haber–Bosch technology for operation on a smaller scale,^28^ ammonia production below 240 tonnes per day is not expected to be economically competitive.^17^

The Haber–Bosch process is a commercially mature technology, but with the evolving environment and energy landscape, there is a pressing need to establish alternate methods for ammonia synthesis on the industrial level. Firstly, from an environmental perspective, the HB process is exclusively responsible for over 1.5% of the global CO_2_ emissions, which amounts to approximately 350 million tonnes per year.^29,30^ As alluded to earlier, this is primarily because the hydrogen for ammonia synthesis is almost exclusively fossil fuel derived. Replacing natural gas with hydropower-electrolysis as the hydrogen source, making so-called ‘green ammonia’ (Fig. 1), would reduce CO_2_ emissions by an estimated 75%.^31^ This transition is seen as being indispensable to meeting the Paris Agreement's greenhouse emissions target by 2050.^32,33^

Secondly, from the standpoint of energy consumption, the conventional method of ammonia synthesis is so energy-intensive that it accounts for over 1% of the global energy consumption.^34^ Replacing coal with methane to produce hydrogen and improvements in compressor and energy integration technologies have enabled significant reductions in the energy consumption for ammonia synthesis, down from over 60 GJ per tonne-NH_3_ in the 1950s to around 30 GJ per tonne-NH_3_ in the present day.^15^ On selecting optimum values for process parameters, such as recycle ratio, inert level, separator temperature, *etc.*, the energy consumption for the ammonia synthesis loop has been found to depend strongly on the equilibrium temperature at the reactor exit.^35^ This analysis estimates that reducing the operating temperature from 713 K to 633 K would result in an energy saving of around ∼1 GJ per tonne-NH_3_.^35^ Catalysts that enable operation at a lower temperature can effectively lower the process pressure, thereby bringing down the compression energy, which contributes significantly to energy costs.^29^ The correlation of the theoretical ammonia yield with reaction temperature and pressure demonstrates that with a highly active catalyst, the single-pass ammonia yield under milder reaction conditions (temperature < 673 K and pressure < 50 bar) can surpass the yields typically achieved in a state-of-the-art Haber–Bosch process.^36^ However, considerations of the effects of milder reaction conditions are rarely as simple as they might appear on first glance. For example, the transition towards a lower process pressure must take into consideration the lower temperature that would be required in the separator downstream for ammonia recovery. Since refrigeration for product condensation is typically achieved by cooling with ammonia, additional refrigeration duty would be required if temperatures below 248 K are needed.^35^ There is a growing research emphasis on replacing the condensation technology with ammonia absorption in sorbents, such as metal halides.^37,38^ This approach can separate ammonia at much lower partial pressures and thereby, further improve the energy efficiency of a low-pressure process.

Reaction systems that enable ammonia synthesis under milder conditions are also expected to be scale-flexible, which will make them a more economically viable alternative to the conventional Haber–Bosch process for downscaled green ammonia production. Economic feasibility studies of offshore wind-powered ammonia manufacture and energy storage systems show that the Haber–Bosch ammonia synthesis loop accounts for between 20 to 25% of the total system capital cost.^17,39,40^ Since much of this cost is driven by the high pressure of the synthesis, catalysts that facilitate efficient synthesis under milder conditions are expected to reduce capital costs and improve the economic viability of decentralized small-scale ammonia production. This is particularly important having established the emphasis on transition from fossil fuel-based to renewable electricity-driven hydrogen production. As opposed to processes that produce hydrogen from fossil fuels, water electrolysis units using renewable electricity do not significantly benefit from economies of scale and their modular nature makes them more suited to small-scale operation.^15,17^ A modularized ammonia synthesis process also presents other advantages, such as faster response to tackle intermittency associated with renewable electricity sources^15^ and simpler unmanned operation.^28^

Ammonia's global demand continues to increase every year, primarily because of its use in agriculture and refrigeration systems. However, for an ammonia supply chain to be established in the global energy/transportation sector, the production would have to be increased manifold. As an example, in maritime transportation, where ammonia is under active consideration as a zero-carbon fuel,^41^ replacement of the energy content from existing fuels with ammonia (at similar conversion efficiencies) would require all of the current world ammonia production. Therefore, small-scale decentralized facilities for ammonia manufacture provide a means to cater to the growing product demand through a less energy-intensive process having a lower ecological footprint. Pathways for ammonia synthesis under milder conditions are based on (i) thermal heterogeneous catalysis, (ii) electrochemical nitrogen fixation, and (iii) plasma-assisted catalysis (Fig. 1). Each of these routes are at different points in their research and development trajectory. Substantial literature is now available on the electrochemical reduction of nitrogen using an aqueous electrolyte, but order-of-magnitude increases in ammonia selectivity and reaction rates, along with significant reductions in required overpotential are needed before commercial systems are likely.^42,43^ Indeed, the low reaction rates have resulted in ambiguity around the nature of ammonia detected in some experiments, with recent benchmarks for testing with isotopically-labelled nitrogen now established to provide greater confidence in the data.^44,45^ Likewise, the low energy efficiency of plasma-stimulated reaction systems remains a concern.^46,47^ Given the urgency of the decarbonization trajectory required to meet the goals of the Paris Agreement, this perspective deals exclusively with the more technologically mature approach of using thermal heterogeneous catalyst systems for ammonia synthesis. More information on the electrochemical and plasma-assisted routes can be found elsewhere.^42,46,48^

The objective of this article is to identify the potential of new-generation heterogeneous catalysts to enable small-scale ammonia synthesis under milder reaction conditions. While industrial catalysts for HB synthesis haven't evolved dramatically since their initial optimization, numerous interesting approaches to catalyst design for low-temperature ammonia synthesis have emerged in the academic literature over the last decade. This makes it an opportune moment to review the progress and identify performance and knowledge gaps that need to be bridged going into the future. We begin by assessing the catalytic performance of different materials that have been reported in published literature thus far and present the major takeaways from the data collation exercise, categorizing important conceptual advances and catalyst improvement strategies. Building on this analysis, we benchmark state-of-the-art catalyst performance against industrial viability criteria and proceed to comment on key catalyst performance metrics that next-generation catalysts must meet to realize economically viable, small-scale, green ammonia production. We also provide a perspective on how a multifaceted approach in catalyst design and testing can pave way for improved performance in the future.

## Focus on catalyst performance

In the following sections, we scrutinize the performance of different heterogeneous catalysts in ammonia synthesis, highlight the key principles and factors that dictate their performance, and propose strategies to achieve higher catalytic activity under milder conditions. Iron-based catalysts, such as magnetite and wüstite, constitute the first-generation of Haber–Bosch catalysts, modified forms of which remain in industrial use to date. Interestingly, a catalyst's intrinsic activity is not the critical factor in influencing the choice of the catalyst under Haber–Bosch synthesis conditions; instead, catalyst cost, tolerance to poisons (considering natural gas is the most common hydrogen source) and ease of regeneration are among the more important criteria.^17,49^ At the high temperatures and pressures that characterize the HB process, approaching a single-pass conversion that is close to the thermodynamic equilibrium is not difficult even with slightly less active catalysts.^17,49^ However, the same does not hold for ammonia synthesis at lower temperatures and pressures, wherein a highly active catalyst is paramount to achieve satisfactory performance. At a pressure of 10 bar and a weight hourly space velocity (WHSV) of 12 000 mL g_cat_^−1^ h^−1^ (H_2_/N_2_ = 3 : 1), an iron-based commercial HB catalyst yields an ammonia productivity of approximately 1900 μmol g^−1^ h^−1^ at 700 K, which falls to 600 μmol g^−1^ h^−1^ at 600 K.^50^ However, the corresponding equilibrium ammonia composition increases from around 2 mol% at 700 K to 10 mol% at 600 K for an operating pressure of 10 bar. Therefore, the decreased productivity of the HB catalyst at lower temperatures, despite the increase in the thermodynamically permissible ammonia concentration, establishes the necessity to explore novel catalysts for use under milder reaction conditions.

**Fig. 2:**
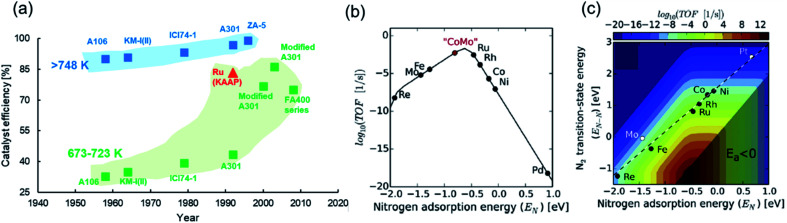
(a) Catalyst efficiencies of different iron-based industrial HB catalysts at pressures above 100 bar and temperatures above 748 K (blue scatter points) and temperatures between 673 K and 723 K (green scatter points). For comparison, the efficiency of the ruthenium catalyst (KAAP HB process) at 698 K is shown in red. A106, KM-I(II) and ICI74-1 catalysts are Fe_3_O_4_-based; A301, ZA-5 and FA400 are Fe_1−*x*_O-based. Data for catalyst efficiencies collated from the following ref. 17 and 59–61; (b) volcano-type relationship between catalyst activity and nitrogen adsorption energy for different transition metals.^55^ Reproduced from ref. 55 with permission from Elsevier, copyright 2015; (c) catalyst activity as a function of nitrogen adsorption energy and N_2_ transition-state energy with the linear scaling relation exhibited by transition metals shown as a dashed line.^55^ Reproduced from ref. 55 with permission from Elsevier, copyright 2015.

The poor performance of commercial HB catalysts at lower temperatures can also be realized in terms of ‘catalyst efficiency’ (Fig. 2a), which we define as the ratio of outlet ammonia concentration to the equilibrium ammonia concentration under reaction conditions in a single pass. An efficiency approaching 100% is indicative of a catalyst delivering an ammonia yield close to the thermodynamic upper bound. Ammonia synthesis catalysts at pressures greater than 100 bar and temperatures exceeding 748 K have historically had respectable efficiencies, with more recent developments now enabling efficiencies very close to 100% (Fig. 2a). However, for temperatures between 673 K and 723 K, and pressures greater than 100 bar, which are still far from ‘mild’ reaction conditions, the discovery of catalysts with high efficiencies have only come about in the last couple of decades (Fig. 2a). This uptick in performance is largely attributed to the switch from magnetite-based to wüstite-based catalysts and the use of electronic promoters (*vide infra*). On the whole, Fig. 2a shows that enabling high catalyst activity at lower temperatures has been a significant challenge, and as demonstrated earlier, at a temperature and pressure of 600 K and 10 bar, respectively, HB catalyst performance is far from satisfactory. In order to address catalyst development strategies for mild-condition ammonia synthesis, we briefly touch upon a few fundamental mechanistic considerations below.

## Designing ammonia synthesis catalysts

Heterogeneously-catalyzed ammonia synthesis typically comprises the following steps: (i) dissociative adsorption of nitrogen and hydrogen, (ii) reaction of adsorbed N and H species to yield surface-bound ammonia, and (iii) desorption of ammonia. These steps constitute the more widely studied Langmuir–Hinshelwood model for ammonia synthesis; however, a smaller class of catalysts have also been shown to synthesize ammonia through the vacancy-mediated Mars–van Krevelen and associative adsorption mechanisms (*vide infra*). With most of the conventional ammonia synthesis catalysts, the dissociative adsorption of nitrogen, characterized by a high activation barrier, happens to be the rate-limiting step.^51–53^ This translates in the experimentally observed reaction order in N_2_ being close to unity. The difficulty in nitrogen dissociation necessitates the severe reaction conditions of HB synthesis and explains the poor performance of catalysts at lower temperatures. For such reactions, the Sabatier principle has long been used to guide decisions on catalyst choice, which describes the optimum catalyst as one that binds the relevant atoms/molecules with an intermediate strength.^54,55^ In the context of ammonia synthesis, the ideal catalyst would be one that adsorbs nitrogen strongly enough in order to be able to activate it, but weakly enough to allow for the desorption of intermediates and ammonia. This results in a volcano-type relationship between catalyst activity and nitrogen adsorption energy (Fig. 2b), where transition metals (TMs) on the left, such as Mo, perform poorly because of a very strong adsorption of nitrogen, while those on the right, such as Co, result in an inferior performance because of a weak interaction with nitrogen. However, a CoMo alloy is postulated to be closer to the theoretical optimum (Fig. 2b), and experimental work confirms that the activity of the bimetallic catalyst is superior to iron catalysts at 573 K and 50 bar.^56^ The volcano plot also explains the higher activity observed with second-generation ruthenium-based HB catalysts in comparison to the iron-based materials. While ruthenium helps realize high catalyst efficiencies under HB conditions (Fig. 2a), its commercial use is limited due to its higher cost compared to iron^57^ and susceptibility to hydrogen poisoning at high pressures.^58^

Staying with the Sabatier principle, the volcano-type relationship can be formulated as a linear scaling relationship between the N_2_ transition-state energy, which is representative of the activation barrier for nitrogen dissociation, and the adsorption energy of nitrogen (and, by extension, NH_*x*_ intermediates^62^) for various transition metals.^55,63^ In other words, TMs that are efficient at activating nitrogen also bind strongly with intermediate species, while those that weakly bind with the intermediates are poor at activating N_2_. Nørskov *et al.* combined the scaling relationship with a kinetic model to map the catalyst turnover frequency (TOF) onto the descriptor space (Fig. 2c), showing the maximum catalyst activity (dark red spot) to be well adrift from the scaling line traced by TMs.^55,57^ This reveals both the considerable scope for improvement in catalyst development, and the limitation of alloying approaches in achieving this improvement. Hence, the search for better ammonia synthesis catalysts is often perceived to be synonymous with the quest for materials that tackle nitrogen dissociation and intermediate adsorption more efficiently.

The first set of approaches targeting higher catalyst activity can be classified as attempts to ‘shift’ the scaling relation towards the hotspot of the activity map. Consider the addition of alkali metal oxides, such as K_2_O, to conventional iron catalysts, which results in higher TOFs under HB conditions.^64,65^ Such additives are called ‘electronic promoters’ since they increase the rate of dissociative nitrogen adsorption by modifying the electronic properties of the catalyst surface. Being an electron donor, K_2_O enriches the available electron density at the iron surface, which in turn enhances the back donation of electron density from the TM to the antibonding π-orbitals of nitrogen, thereby promoting easier nitrogen dissociation.^66,67^ Hence, the potassium promoter does not significantly alter the adsorption energy of nitrogen onto the metal surface but enables a higher catalytic rate by virtue of facilitating nitrogen dissociation. Since the promoter's role is primarily to reduce the activation barrier for nitrogen dissociation, its use results in a favourable downward shift of the scaling relation, thereby unlocking superior catalyst performance.^55^

The same approach of electronic promotion has also been extended to ruthenium-based catalysts, where supporting ruthenium on electrides, such as C12A7:e^−^,^58^ LaScSi,^68^ and Y_5_Si_3_,^69^ ameliorates the reaction rate under mild conditions. Being materials with cavity-trapped electrons, electrides have a high electron donor ability and a low work function that help expedite nitrogen dissociation on ruthenium. Such ‘support effects’ are not just observed with electrides but also with alkaline earth metal–nitrogen–hydrogen (M–N–H) materials. The turnover frequency of Ru/Ca(NH_2_)_2_ is superior to that of Ru-loaded C12A7 electrides and one of the reasons for the impressive catalytic activity is the high electron donor ability calculated for the Ru/Ca(NH_2_)_2_ interface.^70^ A further pronounced increase in activity is seen when a barium-doped Ca(NH_2_)_2_ support is used. The pre-treatment of this catalyst under hydrogen results in the formation of Ba(NH_2_)_2−*x*_, which has a higher electron donating ability than Ca(NH_2_)_2_ and results in greater activity.^71^ Furthermore, since M–N–H materials and electrides can reversibly store hydrogen, they offer an elegant solution to the challenge of hydrogen poisoning, an undesired phenomenon that TMs such as ruthenium are liable to under reaction conditions.^72,73^ Besides M–N–H materials and electrides, hydrogen poisoning can also be countered by modifying conventional supports. The use of an electrostatically polar MgO(111) in place of a standard nonpolar MgO to support ruthenium facilitates the migration of adsorbed H species from the ruthenium surface to the O^2−^ sites, thereby alleviating the issue of hydrogen poisoning.^74^ Likewise, increasing the calcination temperature of an alumina support from 1073 to 1573 K results in multiple phase transformations that minimize hydrogen poisoning and decrease the activation energy for ammonia synthesis.^75^ In the case of ceria-supported ruthenium catalysts, diverse catalyst pre-treatment strategies, including CO activation,^76^ N_2_H_4_ reduction,^77^ and NaBH_4_ treatment,^78^ have been found to have a profound effect on ammonia synthesis activity. These treatments enhance the electronic metal–support interaction and increase the fraction of metallic and exposed ruthenium, all of which have a positive effect on nitrogen activation. In addition, these treatments also weaken the inhibition effect of adsorbed hydrogen species in the catalysis. The effectiveness of such strategies that combine considerations of nitrogen and hydrogen adsorption/desorption is discussed in greater detail in the next section (*vide infra*).

**Fig. 3:**
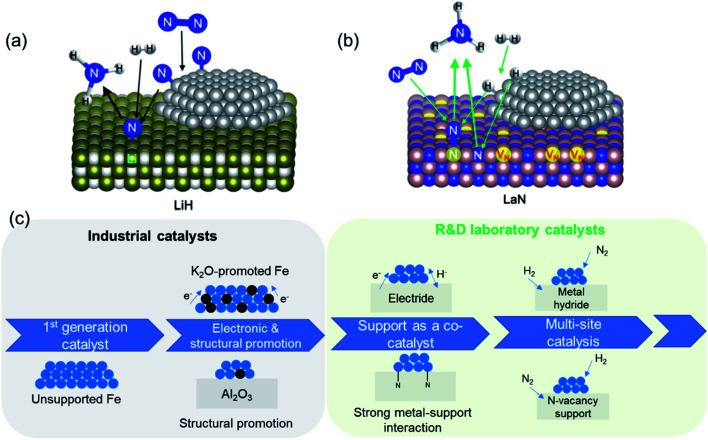
(a) Schematic of multi-site reaction mechanism over transition metal-loaded LiH; reproduced from ref. 84 with permission from Springer Nature, copyright 2020; (b) schematic of Mars–van Krevelen reaction mechanism over transition metal-loaded LaN; reproduced from ref. 84 with permission from Springer Nature, copyright 2020; (c) evolution in the complexity of heterogeneous catalyst design for ammonia synthesis: from a simple metal surface to multi-site approaches.

The second set of approaches for improving catalyst activity encompass attempts to ‘break’ the scaling relationship governing ammonia synthesis. As described earlier, the transition-state energy for ammonia synthesis scales with the adsorption energy of nitrogen on TMs. However, to optimize catalyst performance, it would be desirable to manipulate the two variables independently, and thus allow for strong reactant activation and weak binding to intermediates simultaneously.^55,63^ This can be achieved in a ‘multi-site’ or relayed catalytic sequence, wherein different active sites are responsible for nitrogen dissociation and the subsequent hydrogenation to NH_3_ (Fig. 3a). This approach echoes the catalytic function of some enzymes, including proposed reaction mechanisms for nitrogenase, where the active site is hypothesized to change during the reaction (*e.g.* through altered coordinating groups) to better optimize individual reaction steps.^79–81^ Chen *et al.* proposed the use of transition metal–lithium hydride composite catalysts for this purpose, where the TM is responsible for activating nitrogen and LiH facilitates the removal of the activated nitrogen species from the TM and subsequent conversion to NH_3_.^82^ The introduction of LiH to the iron catalyst augments the ammonia synthesis rate by more than an order of magnitude at 573 K and 10 bar. Under the same reaction conditions, where other TMs, such as manganese and cobalt, are barely active, compositing with LiH results in a catalytic performance on par with that of the Fe–LiH composite.^82^ Likewise, a two active-site model has also been posited for the synthesis of ammonia over ternary intermetallic LaCoSi, where the activated hydrogen on LaCoSi is envisioned to serve as the second active site that extracts activated nitrogen from cobalt.^83^ The catalytic productivity of LaCoSi is as much as 60-fold higher than of conventional supported cobalt catalysts.^83^ As explained in the examples cited above, the ‘multi-site’ approach to breaking the scaling relation is where the second site facilitates the extraction of activated nitrogen from the first site, which is typically a TM. An alternate dual active-site model to circumvent the scaling relationship is based on the vacancy-enabled activation of nitrogen. Ye *et al.* illustrated this through a Ni/LaN catalyst, wherein the nitrogen vacancies in the nitride support efficiently activate nitrogen, while the TM is chosen for its excellent hydrogen activation properties (Fig. 3b).^84^ This makes it fundamentally different from the examples discussed earlier, as the TM's primary role has changed from nitrogen activation to hydrogen activation; nevertheless, these catalyst systems are unified by the approach to spatially separate nitrogen and hydrogen activation in order to overcome the constraint set by the scaling relationship. In such nitride and other oxynitride–hydride supports, isotopic nitrogen exchange and ammonia synthesis experiments reveal the participation of lattice nitrogen in the catalysis.^85,86^ Hence, ammonia synthesis in these materials occurs through the Mars–van Krevelen (MvK) mechanism, where nitrogen activation is mediated by the nitride/anionic vacancies,^87,88^ distinguishing it from the more classical dissociative adsorption of nitrogen that occurs over transition metals. In the case of Co/CeN and Co_3_Mo_3_N, an associative adsorption mechanism is also proposed, where hydrogenation of adsorbed molecular nitrogen (and not dissociated nitrogen) yields ammonia.^89,90^ Hence, adopting strategies to ‘break’ the scaling relation can enable considerable improvements to catalyst performance at low temperatures and pressures, which are particularly promising to realize more efficient small-scale, mild-conditions ammonia synthesis. Alongside the constraint imposed by the scaling relationship, the use of milder conditions for ammonia synthesis can present a challenge for nitrogen dissociation. As elucidated above, multi-site catalysts and vacancy-enabled nitrogen activation are two ways of addressing the issue. Another interesting strategy is hydrogen-assisted nitrogen activation. For example, the rate of nitrogen fixation in a Ta_3_N_3_H^−^ cluster was found to be considerably higher than in dehydrogenated Ta_3_N_3_^−^. The presence of hydrogen improves the reactivity of the former cluster by decreasing the nitrogen adsorption energy and storing more electrons in the Ta–Ta bond.^91^ In another study that investigated molybdenum-catalyzed ammonia production in the presence of samarium diiodide and an alcohol, the high productivity was ascribed to a proton-coupled electron-transfer process that is enabled by the weakening of the alcohol O–H bond coordinated to SmI_2._^92^ Beyond heterogeneous catalysis, the hydrogen-assisted nitrogen activation pathway is also of great interest in electro- and photocatalysis.^93,94^

Thus far, we looked at approaches to enhance ammonia synthesis rates by improving the active site(s)-turnover frequency, specifically addressing the issues of nitrogen dissociation and intermediate adsorption energy. In other words, these approaches are aimed at achieving a higher intrinsic activity. However, along with the intrinsic activity of an active site, catalyst performance is also a function of how the active sites are hosted in a heterogeneous catalyst and ‘presented’ to reaction conditions. This includes considerations of ‘physical factors’, such as active site dispersion, surface area of the material, spectator site concentration, and effects of porosity on catalyst performance, among others. Historically, even with iron-based HB catalysts, structural promoters, such as Al_2_O_3_ and MgO, have been added to the catalyst formulation to increase the surface area of the catalyst and the iron dispersion.^95^

We now direct our focus to examine these aspects in the context of recently reported catalysts for ammonia synthesis. While the use of electrides as a catalyst support for ammonia synthesis is primarily because of its electron donor ability, these materials tend to have extremely low specific surface areas (typically 1–2 m^2^ g^−1^), which translates into a low dispersion of the active TM. Selective etching of electrides, such as LaRuSi and CeRuSi, using EDTA results in a nearly 3-fold increase in the specific surface area of the material and a commensurate increase in catalytic productivity.^96^ It is worth adding that the EDTA treatment does not change the catalyst TOF, meaning the intrinsic activity of the catalyst is not altered, but the increased productivity is because of the exposure of a greater fraction of ruthenium on the catalyst surface. Likewise, novel synthesis of electrides have also been reported to produce mesoporous versions of the material with better mass transport properties for ammonia synthesis catalysis.^97^ Similarly, H_2_ pretreatment of a Ba–Ca(NH_2_)_2_ support converts it into a mesoporous structure with a high surface area.^71^ Such ‘tailored synthesis’ strategies help develop catalysts with a high density of active sites that are easily accessible under reaction conditions. Another example is the impressive performance of the Co–N–C catalyst system, which is largely ascribed to pyrrolic nitrogen serving as an anchor to yield atomically dispersed Co–N_*x*_ sites.^98^ Next, let us consider the case of *n*BaH_2_–*x*%Co/CNT catalysts, where *n* is the molar ratio of Ba to Co and *x*% is the mass ratio of Co to CNT. Activity data for a range of catalysts synthesized with 1 ≤ *n* ≤ 5 and 5 ≤ *x* ≤ 20 shows the presence of an optimum in catalyst performance in both variables, with 3BaH_2_–10%Co/CNT yielding the highest productivity.^99^ Such screening of catalyst compositions can guide synthesis protocols in finding the optimal active site dispersion and loading, particularly in cases similar to the above example, where a support hosts both a catalyst and a co-catalyst. ‘Tailored synthesis’ includes undertaking steps to produce specific desired shapes of catalyst particles, such as flat-shaped Ru nanoparticles with a narrow distribution^70^ or Ru–Ba core–shell nanoparticles,^71^ both of which help improve catalytic activity in low-temperature ammonia synthesis. Hence, different aspects of material engineering need to complement the design of active sites with intrinsically high TOF for catalysts to have an impressive performance.

The developments reported in the preceding discussion represent the evolution in the design of ammonia synthesis catalysts and their supports, which we summarize schematically in Fig. 3c. This charts the progression from identification of active transition metal catalysts to the “doubly promoted” catalysts used in industry today and the more complex support architectures and multi-site catalysts being developed in R&D laboratories.

## Assessing recent catalyst developments

Table 1 collates the kinetic data of promising heterogeneous catalysts that have been reported for ammonia synthesis under milder conditions. In addition, for each of these materials, we identify the reason(s) for the better catalytic performance in comparison to conventional HB catalysts. In line with the discussion presented in this section, Table 1 ascribes improved catalytic activity to one or a combination of the following reasons: electronic promotion effects, support effects, multi-site catalysis including vacancy-mediated MvK catalysis, physical factors and tailored synthesis. The kinetic parameters of standard HB catalysts are included at the top of the table for comparative purposes. We see that most of the new generation catalysts have successfully addressed the bottleneck of nitrogen activation in ammonia synthesis. A reaction order in N_2_ significantly smaller than unity shows that the dissociative adsorption of nitrogen is no longer the rate-determining step in these catalyst systems. Likewise, ruthenium supported on electrides and M–N–H materials report positive reaction orders in H_2_, which is in contrast to conventional supported Ru catalysts. This reflects the success of using reversible hydrogen stores as a support to negate hydrogen poisoning of the catalyst during ammonia synthesis. Interestingly, most of the catalysts converge on an apparent activation energy in the range of 45–60 kJ mol^−1^ and a negative reaction order in NH_3_, typically between −1 and −1.5. With regards to the latter, an emphasis on facilitating ammonia desorption from the catalyst surface is worth considering as a novel research direction for the future.

**Table 1:** Summary of kinetic parameters of promising heterogeneous catalysts reported for ammonia synthesis under mild conditions and classification of reasons for improved catalyst performance^a^

Catalyst	Kinetic parameters						Reasons for improved catalyst performance					Ref.
	N_2_ order	H_2_ order	NH_3_ order	*E*_a_ (kJ mol^−1^)	Temp. (K)	Press. (bar)	Electronic promotion effects	Support effects	Multi-site catalysis/MvK mechanism	Physical factors (surface area, porosity *etc.*)	Tailored synthesis	
**Standard Haber–Bosch catalysts & conventionally supported TM catalysts**												
Fe KM1 catalyst	0.9	2.2	−1.5	70	593–673	10						58
Ru/MgO	0.82	−0.38	−0.68	n. a	593–673	1						
Cs–Ru/MgO	1.0	−1.2	−0.1	124	523–613	10						

**Electride-supported catalysts**												
Ru/C_12_A_7_:e^−^	0.46	0.97	−1	49	593–673	1	✓	✓	✗	✗	✗	58 and 100
Ru/Ca_2_N:e^−^, Ru/Ca_2_NH	0.53	0.79	−1.03	60	533–613	1	✓	✓	✗	✗	✗	72 and 101
Ru/Y_5_Si_3_	n. a	n. a	n. a	50	593–673	1	✓	✓	✗	✗	✗	69
Ru/C_12_A_7_ (mesoporous)	n. a	n. a	n. a	77–83	573–673	1	✓	✓	✗	✓	✓	97
Co/C_12_A_7_:e^−^	1.08	1.40	−1.18	50	473–613	1	✓	✓	✗	✗	✗	102
Ru/LaScSi	0.53	0.48	−1.03	49	533–673	1	✓	✓	✗	✗	✗	68

**Ternary intermetallics**												
LaCoSi	0.45	0.80	−1.5	42	573–673	1	✓	✗	✓	✗	✗	83
LaRuSi	0.98	0.66	−1.05	40	573–673	1	✓	✗	✓	✗	✗	103
LaRuSi (EDTA treated)	0.93	0.62	−0.85	48	573–673	1	✓	✗	✓	✓	✓	96
Co_3_Mo_3_N	0.86	0.77	−1.43	59	573–673	1	✗	✗	✓	✗	✗	83 and 87

**Ru/M(N)H series**												
Ru/Ca(NH_2_)_2_	0.53	0.55	−1.5	59	553–613	1	✓	✓	✗	✓	✓	70
Ru/CaH_2_	0.55	0.87	−1.11	51	523–613	1	✓	✓	✗	✗	✗	101
Ru/BaO–CaH_2_	0.47	0.45	−1.3	41	543–593	1	✓	✓	✗	✓	✗	73
Ru/Ba–Ca(NH_2_)_2_	0.96	0.75	−0.92	59	533–613	9	✓	✓	✗	✓	✓	71
Ru_2_Y	0.94	0.81	−0.73	73	593–673	1	✓	✗	✗	✓	✓	104
Ru/CaFH	n. a	n. a	n. a	20	323–398	1	✓	✓	✗	✓	✓	36
Ru/BaTiO_2.5_H_0.5_	0.70	0.20	−0.64	57	593–673	50	✓	✓	✓	✗	✗	105
Ru/BaCeO_3−*x*_N_*y*_H_*z*_	1.07	1.00	−0.98	62	533–673	9	✓	✓	✓	✗	✗	85
Ru/La_0.5_Ce_0.5_O_1.75_	0.76	0.15	−0.36	64	573–673	10	✓	✓	✓	✓	✓	106
Ru/La_0.5_Pr_0.5_O_1.75_	0.85	0.53	−0.36	n. a	573–673	10	✓	✓	✓	✓	✓	107
Ru/LaN/ZrH_2_	0.65	0.43	−1.03	64–74	573–673	10	✓	✓	✓	✗	✓	108
Ru/CaCN_2_	0.82	−0.03	−0.54	64	553–613	10	✓	✓	✓	✗	✗	109
Ru/Ti–Ce oxide	n. a	n. a	n. a	76	603–673	10	✓	✓	✗	✓	✓	110
Ru/silicalite-1	0.15	0.36	n. a	55	548–648	1	✗	✓	✗	✓	✓	111

**Other TM-M(N)H & TM/LaN catalysts**												
Cr–LiH	0.43	0.62	−1.2	64	498–598	10	✗	✗	✓	✗	✗	82
Mn–LiH	0.12	1.1	−1.3	51	498–598	10	✗	✗	✓	✗	✗	82
Fe–LiH	0.37	0.88	−1.3	47	498–598	10	✗	✗	✓	✗	✓	82 and 112
Co–LiH	0.48	0.65	−1.2	52	498–598	10	✗	✗	✓	✗	✗	82
Mn_4_N–BaH_2_	0.35	n. a	n. a	64	548–623	10	✗	✗	✓	✗	✗	113
Mn_4_N–LiH	n. a	n. a	n. a	60	548–623	10	✗	✗	✓	✗	✓	113
Co–BaH_2_/CNT	0.43	0.58	−1.27	58	523–623	10	✗	✓	✓	✗	✓	99
Ni–BaH_2_	Tested in chemical looping process			47 (nitrog.)	463–533 (nitrog.)	1	✗	✗	✓	✗	✗	114
				34 (hydrg.)	373–473 (hydrg.)							
Ni–BaH_2_/Al_2_O_3_	Chemical looping process			n. a	Same as previous	1	✗	✓	✓	✓	✗	114
Fe/BaTiO_3−*x*_H_*x*_	0.56	0.75	−1.1	54	573–673	50	✓	✓	✓	✗	✗	105
Co/BaTiO_3−*x*_H_*x*_	n. a	n. a	n. a	69	573–673	50	✓	✓	✓	✗	✗	105
Fe/BaCeO_3−*x*_N_*y*_H_*z*_	1.05	1.67	−1.34	44	533–673	9	✓	✓	✓	✗	✗	85
Co/BaCeO_3−*x*_N_*y*_H_*z*_	0.96	2.11	−1.15	48	533–673	9	✓	✓	✓	✗	✗	85
Rb_2_ [Mn(NH_2_)_4_]	n. a	n. a	n. a	54	473–623	10	✗	✗	✓	✗	✓	115
K_2_ [Mn(NH_2_)_4_]	n. a	n. a	n. a	57	473–623	10	✗	✗	✓	✗	✓	115
Cs-FePc	0.75	2.08	−2.16	41	523–673	10	✓	✓	✗	✓	✓	50
Ba-CoPc	0.87	1.92	−0.21	61	523–673	10	✓	✓	✗	✓	✓	50
Co–N–C	n. a	n. a	n. a	50	513–633	10	✗	✓	✓	✓	✓	98
Ni/LaN NPs	1.2	1.2	−1.7	58	573–673	1	✗	✓	✓	✓	✓	84
Ni/LaN bulk	1.1	1.4	−1.8	60	573–673	1	✗	✓	✓	✗	✗	84
Ni/CeN NPs	1.2	1.6	−1.4	54	573–673	1	✗	✓	✓	✓	✓	86

a Catalytic experiments are performed using a feed gas composition of 1 : 3 N_2_ : H_2_ and chemical looping experiments use sequential flows of pure nitrogen and hydrogen.

**Fig. 4:**
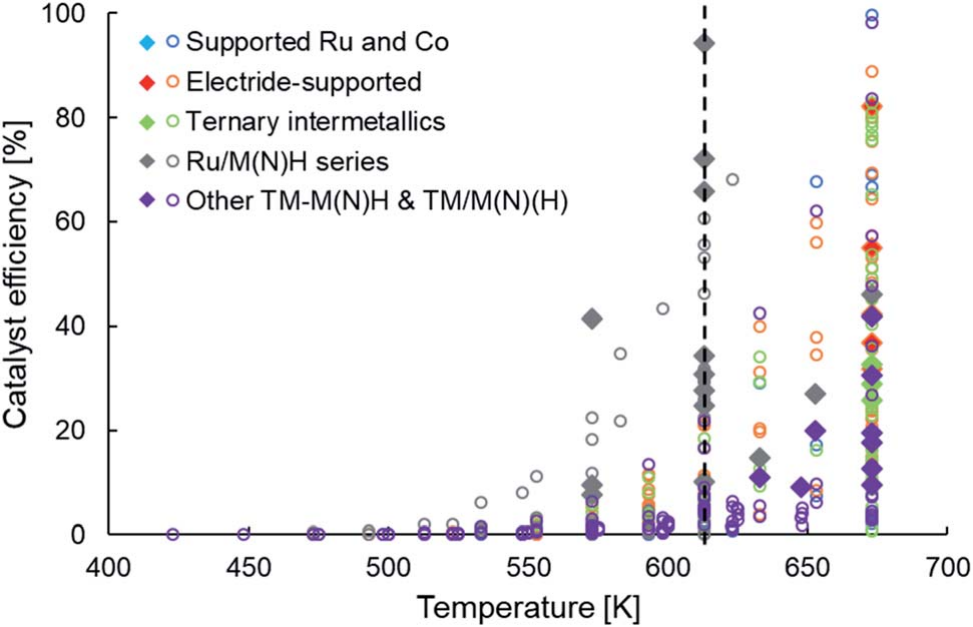
Scatter plot showing the efficiencies of various types of heterogeneous catalysts as a function of temperature for ammonia synthesis at pressures no greater than 10 bar. The open circles and filled diamonds are used to represent catalyst productivities lower and greater than 10 mmol NH_3_ per g_cat_ per h, respectively. For categorization of catalyst types and references for catalytic activity data, please refer to Table 1.

The newer classes of ammonia synthesis catalysts, as categorized in Table 1, have enabled greater ammonia productivities under milder conditions when compared to HB catalysts. However, unless we return to the metric of catalyst efficiency introduced earlier, it is difficult to judge the performance of these systems and identify the performance gaps that remain. Unlike catalyst productivity, catalyst efficiency factors in the effects of temperature and pressure on ammonia yield, making it more appropriate to draw comparisons between systems. Fig. 4 presents the efficiency of these heterogeneous catalysts in a temperature range of 400 K to 700 K at an operating pressure no greater than 10 bar. The filled diamonds in Fig. 4 highlight catalysts that have a productivity greater than 10 mmol NH_3_ per g_cat_ per h. Similar to the trend observed in Fig. 2a, we see that high catalyst efficiencies are accomplished at temperatures greater than 650 K, but at 600 K and lower, the performance is far from satisfactory (Fig. 4). The vertical line drawn at a temperature of 613 K marks the stark contrast in the number of catalysts with reasonable efficiencies on either side of this temperature. Despite taking diverse routes to elevate catalyst performance, as delineated earlier and classified in Table 1, the vast majority of the newer generation catalysts show a pronounced drop in efficiency on lowering the temperature from 673 K to 613 K. A close inspection of these catalysts and the outliers offers important lessons that can guide future catalyst design. While some ruthenium-based catalysts, including Ru/C12A7:e^−^ (ref. 58 and 100) and YRu_2_,^104^ are among materials that follow the trend of sharply declining efficiency with decreasing temperature, falling typically to less than 40% at 633 K, that of Ru/Ca(NH_2_)_2_ and Ru/BaO–CaH_2_ compare more favorably, sustaining an efficiency of over 65% at 613 K. From Table 1, we decipher that the better-performing Ru/Ca(NH_2_)_2_ and Ru/BaO–CaH_2_, not only have a reaction order much smaller than unity for nitrogen but also for hydrogen. This hints at these catalysts being efficient for nitrogen and hydrogen activation. Likewise, another outlier in the general trend of low efficiency at temperatures around 600 K is Ru/CaFH. Alongside Ru/Ca(NH_2_)_2_ and Ru/BaO–CaH_2_, Ru/CaFH is the other catalyst with an efficiency of over 60% at these temperatures, reaching over 90% at 613 K (Fig. 4). In addition to having an excellent electron donor capacity, which would expedite nitrogen dissociation, the CaFH solid solution allows hydrogen desorption at temperatures much lower than observed with other supported ruthenium catalysts.^36^ The presence of F^−^ weakens the ionic bonds between Ca^2+^ and H^−^, lowering the onset temperature for hydrogen desorption in Ru/CaFH to well below 373 K, making it the first heterogeneous catalyst to synthesize ammonia at such temperatures with an activation energy as low as 20 kJ mol^−1^.^36^ In contrast, the main hydrogen-desorption peak in the TPD of electrides, such as C12A7:e^−^ and Ca_2_N:e^−^, are above 673 K.^100,101^ Likewise, the treatment of Ru/CeO_2_ with NaBH_4_ or N_2_H_4_, which results in improved ammonia production rates, shifts the maximum hydrogen consumption peak in a H_2_-TPR experiment to lower temperatures.^77,78^ Similarly, the catalytic performance of ruthenium supported on lanthanide oxyhydrides strongly correlates with the surface hydride ion mobility.^116^ Thus, the common feature of efficient catalysts at low temperatures is that alongside addressing the challenge of nitrogen activation, often through electronic or structural promotion, these materials also have favourable hydrogen adsorption–desorption properties. While strategies that facilitate nitrogen dissociation have enabled recently reported heterogeneous catalysts to surpass activities of conventional catalysts under milder reaction conditions, further improvements in catalyst performance will mostly require the considerations of hydrogen adsorption–desorption to be taken in conjunction with nitrogen activation.

The same proposal can also be extended to other TM catalysts beyond ruthenium. In the discussion on ways to break the scaling relationship in ammonia synthesis, we brought up the case of TM–LiH composites. Among these materials, Co–LiH has a higher activity than the Mn, Fe and Cr analogues at 573 K as well as at 523 K.^82^ Interestingly, the kinetic parameters of the Co–LiH catalyst (Table 1) are very similar to that of Ru/Ca(NH_2_)_2_ and Ru/BaO–CaH_2_ cited earlier. With these TM–LiH composites, LiNH_2_ is proposed to be formed under reaction conditions as a result of LiH abstracting the nitrogen that is activated on the transition metal.^82^ The subsequent hydrogenation of the LiNH_2_ species to NH_3_ is posited as the rate-limiting step in the catalytic cycle. Importantly, the hydrogenation kinetics varies with the choice of the transition metal as the shape of the H_2_-TPR profiles of post N_2_-treated TM-LiH samples are neither identical to each other nor to that of neat LiNH_2_. Furthermore, while the hydrogenation of neat LiNH_2_ has a reaction order in hydrogen of close to unity, ammonia synthesis over Co–LiH has a hydrogen reaction order of less than 0.5 (Table 1). The importance of hydrogenation kinetics in catalyst performance can also be realized from the greater enhancement in the activity of Mn nitride on compositing it with BaH_2_ instead of LiH.^113^ While Mn_4_N–LiH has a smaller reaction order in nitrogen than Mn_4_N–BaH_2_ (Table 1), nitrogen dissociation is no longer the rate-determining step in the catalysis and the difference in catalyst activity stems from the difference in rate of hydrogenation of [LiNH] and [BaNH] species. Analogous to the Ru/CaFH catalyst discussed in the preceding paragraph, hydrogen evolution occurs at relatively low temperatures in the TPD profile of Mn_2_N–BaH_2_, lower by about 50 K in comparison to Mn_2_N–LiH.^113^ These observations reinforce the necessity to assign due importance to the hydrogen-related aspects of the ammonia synthesis catalytic cycle. Based on the data collated and the discussion presented herein, we conclude that catalysts that holistically address nitrogen activation, hydrogen activation, and hydrogenation of adsorbed nitrogen species tend to outperform catalysts designed with a myopic view of expediting nitrogen dissociation.

Staying with non-ruthenium TM-based catalysts, Fig. 4 shows that the efficiencies reported with TM–M(N)H and TM/M(N)(H) materials (where M = group I/II metal, La or Ce) are mediocre and significantly lower than the efficiencies obtained with Ru/M(N)H type catalysts, particularly at temperatures around 600 K. This constitutes an important performance gap that future developments must attempt to bridge. Nevertheless, many of these catalyst systems yield a productivity in excess of 10 mmol NH_3_ per g_cat_ per h, primarily because of the operating pressure being 10 bar (filled purple diamonds, Fig. 4). While CeN-supported Ni approaches 100% efficiency at 673 K and 1 bar pressure, it falls sharply to approximately 40% at 633 K and 20% at 613 K (Fig. 4). Unlike the better performing ruthenium-based catalysts in this temperature regime that were elaborated on earlier, Ni/CeN has high positive reaction orders in nitrogen and hydrogen (Table 1), potentially explaining the observed plummet in efficiency at lower temperatures. In contrast, the TM–LiH systems are at the most 5% efficient, and Co–BaH_2_/CNT is close to 20% efficient at 673 K and 10 bar pressure. Importantly, these catalysts typically report a reaction order in nitrogen of well below unity, and often along with fractional orders in hydrogen. Therefore, their low efficiency is emblematic of a catalyst ‘presentation’ issue, as discussed earlier, and less to do with poor intrinsic activity. Consequently, enhancements in efficiency would have to primarily be driven by changes in synthesis procedures that improve the density, dispersion and accessibility of active sites in these materials. For instance, in the case of Fe–LiH catalysts, a high interfacial area between LiH and Fe particles is paramount for high efficiency. Increasing the molar ratio of LiH to Fe in the catalyst results in smaller Fe particles and greater interfacial surface area between the transition metal and the hydride phase, which translates in better catalyst performance.^112^ Likewise, supporting the Fe–LiH composite on MgO greatly enhances Fe dispersion. As a result, the efficiency of the supported catalyst at 573 K is 30% greater than that of the unsupported version.^112^ For these very reasons, Co–BaH_2_ supported on carbon nanotubes emerges as one of the more efficient catalysts in this category. Exploring porous, high surface area catalyst supports and synthesis strategies that enhance the synergy between the transition metal and the hydride phase would render substantial improvements in efficiency possible. However, even without such supports, a similar outcome could be achieved by engineering the physical structure of the hydride phase, a research direction that is yet to be explored extensively.

Another promising approach to improve the efficiency of such dual active-site materials is to move from a catalytic regime to a looping mode of operation, where nitrogen and hydrogen are flowed sequentially over the material and not simultaneously. For instance, rates of ammonia production with Ni–LiH or Ni–BaH_2_ increase markedly on switching from a catalytic process to a chemical looping protocol.^114^ While the earlier discussion on catalyst active site ‘presentation’ was largely restricted to material engineering aspects, the spatial separation of nitrogen and hydrogen flow can be envisioned as a way of manipulating how the active sites are ‘presented’ to reaction conditions. When Ni–LiH or Ni–BaH_2_ is exposed to a gas mixture of hydrogen and nitrogen, the nickel surface would be preferentially covered with hydrogen, leaving barely any active sites in the composite to activate nitrogen and resulting in low productivity. This issue of undesired competitive adsorption can be averted when the nitrogen fixation and hydrogenation steps are separated; nitrogen is first fixed in the composite material to yield an imide phase, which is hydrogenated in a second step to yield ammonia.^114,117^ A similar two-step pathway for ammonia production has also been identified in composites of manganese nitride and metal (Li or Ba) imides.^118^ The looping mode of operation comes with another advantage of ammonia yield not being bound by a thermodynamic equilibrium upper limit. Therefore, in theory, materials that are more than 100% efficient may be designed; however, this is yet to be demonstrated in practice. Furthermore, a looping mode of operation is expected to be efficient only with materials that undergo a bulk transformation when fixing one or both of the reagents, such as LiH during the fixing of nitrogen in the example mentioned above.

The critical assessment of ammonia synthesis catalysts undertaken in this section helped us appreciate the developments that have been realized thus far and derive important lessons for future catalyst development. We believe that embracing a comprehensive view of catalyst design, where strategies to expedite nitrogen dissociation and facilitate hydrogen adsorption–desorption are considered simultaneously, is vital in the pursuit of more efficient catalysts. Furthermore, such strategies need to be complemented by smart engineering of materials that maximizes active site density and accessibility, and in the case of multi-component catalysts, also maximizes contact between the catalyst and co-catalyst/support. These takeaways are formulated principally from a scientific understanding of the catalysis. However, for the proliferation of small-scale mild-conditions ammonia synthesis facilities worldwide, a perspective on the technoeconomic considerations of catalyst development is equally important. In the next section, we proceed to address catalyst metrics of industrial relevance and extend the catalyst development discussion to cover aspects of economic viability.

## Industrial considerations to catalyst development

A preliminary assessment of the industrial potential of a heterogeneously-catalyzed process can be made on the basis of the following four criteria:^119,120^

• Product selectivity > 70%.

• Product concentration > 3 wt%.

• Catalyst activity of 0.1–10 tonne_product_ per m_reactor_^3^ per h.

• Catalyst consumption of 0.01–1 kg_cat_ per tonne_product_.

While these criteria have been framed for a heterogeneous catalytic process, they can also be extended to analyse chemical looping processes,^121^ such as the one discussed in the previous section.^114^ In the context of this perspective, we are interested in the final two criteria, both of which are defined with a focus on the catalyst, namely the activity and the consumption. Importantly, these catalyst targets were derived from processes for fuels and chemicals that are sold typically from $500 per tonne to over $1000 per tonne.^122^ Since the historic selling price of ammonia is comparable to the lower end of that price range, we take the liberty of using the same performance targets for a preliminary analysis. However, with ammonia costs having rarely gone close to or exceeded $1000 per tonne, the actual activity and consumption thresholds for ammonia synthesis catalysts are likely to be slightly higher and consequently, the values mentioned herein can be safely considered as absolute minimum requirements. Furthermore, for emerging applications of ammonia, such as its use as a hydrogen store and an energy vector, a further reduction in the selling price of ammonia is seen as necessary. For instance, an ammonia cost of $500 per tonne implies a hydrogen cost of ∼$2800 per tonne (without factoring in costs associated with the cracking process), which is higher than current costs of hydrogen produced by steam methane reforming ($1000–2500 per tonne).^123^ Likewise, the same ammonia cost translates to a power cost of $161 per MW per h when used in a combined cycle gas turbine or $100 per MW per h when used in a solid oxide fuel cell, after accounting for the different round trip efficiencies of these systems.^124^ These estimates establish the necessity for the ammonia selling price to fall further, which reinforces the point made earlier that the actual catalyst criteria for process viability are expected to be more stringent than the pre-defined criteria taken up for discussion here. Besides, the economic viability of ammonia synthesis technology hinges on aspects like energy integration and compression efficiency, amongst others. With these caveats in mind, we now proceed to address the catalyst activity and consumption criteria.

**Fig. 5:**
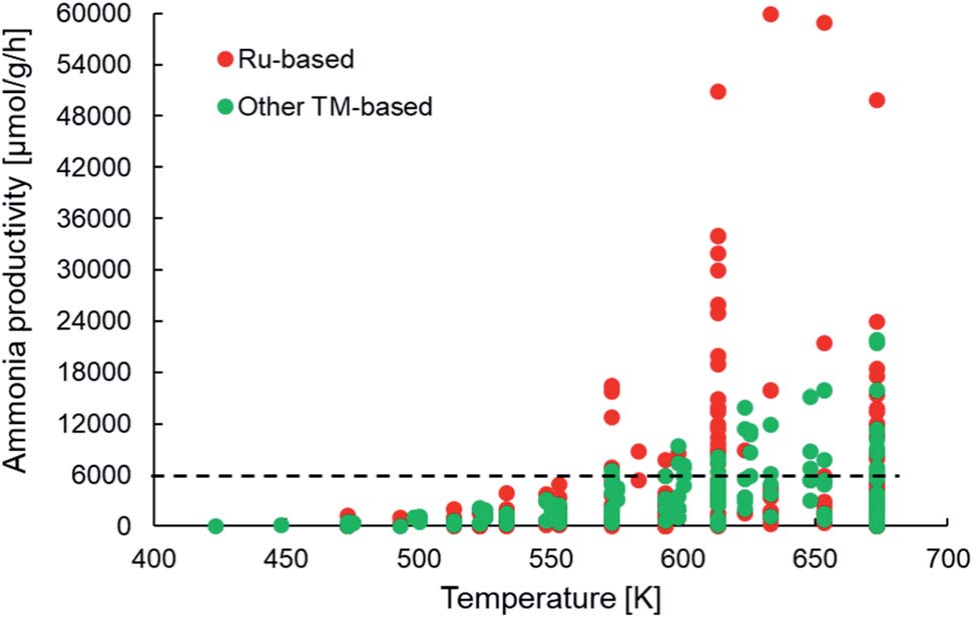
Scatter plot showing the ammonia productivities of various heterogeneous catalysts as a function of temperature for ammonia synthesis at pressures no greater than 10 bar. The same catalyst systems were represented in terms of their efficiency in Fig. 4. References for activity data of the individual catalyst systems can be found in Table 1.

Assuming a catalyst density of 1 tonne per m^3^ for a fixed bed catalytic reactor, the minimum catalyst activity threshold would translate to >0.1 tonne_product_ per tonne_catalyst_ per h or approximately 6000 μmol NH_3_ per g_cat_ per h. From Fig. 5, we observe that many of the newer-generation catalysts indeed fulfill the activity criterion. Most of these catalysts report productivities under 18 000 μmol NH_3_ per g_cat_ per h, with only some Ru-based catalysts yielding productivities above 30 000 μmol NH_3_ per g_cat_ per h. This implies that while many catalysts indeed show an activity greater than the assumed minimum threshold, a vast majority of them surpass it by a small margin, which must be taken into account considering the cost pressure on green ammonia as outlined above. This also has important consequences for the final aspect of catalyst consumption, which is defined as the inverse product of catalyst activity and lifetime. The criterion states that no more than 1 kg of catalyst (or material) should be used to produce 1 tonne of the product. For a catalyst with an ammonia productivity of 12 000 μmol NH_3_ per g_cat_ per h, the consumption criterion can only be met if the material is stable on stream for at least 6 months. To the best of our knowledge, there are no published reports of the newer-generation catalysts being tested over such time periods, and hence, nothing conclusive can be said on whether these materials successfully pass the consumption criterion. However, there are indications that factors such as sensitivity to moisture and the separation and aggregation of the catalyst and co-catalyst phases can adversely affect the stability of some of these materials, especially in electride-supported and TM–M(N)H type catalysts (Table 1) over much shorter timescales.^69,114^ Nevertheless, the important takeaway from this analysis is that many of the newer ammonia synthesis catalysts report productivities closer to the lower bound of the catalyst activity criterion, which consequently requires them to have a reasonably long lifetime in order to meet catalyst consumption targets.

While we have based our analysis thus far on pre-defined industrial performance windows, the aspect of catalyst consumption in ammonia synthesis deserves closer inspection as the cost of the catalysts used in this process tend to vary significantly. On one end of the scale, iron catalysts can cost less than $10 per kg, while those that are ruthenium-based can cost over $500 per kg.^17^ Therefore, we proceed to present a slightly more nuanced analysis of catalyst consumption targets by factoring in the wide variation in material costs. Industrial manufacturing economics state that the catalyst costs are typically a very small percentage of the total manufacturing costs and rarely exceed $10 per tonne of product.^20^

**Fig. 6:**
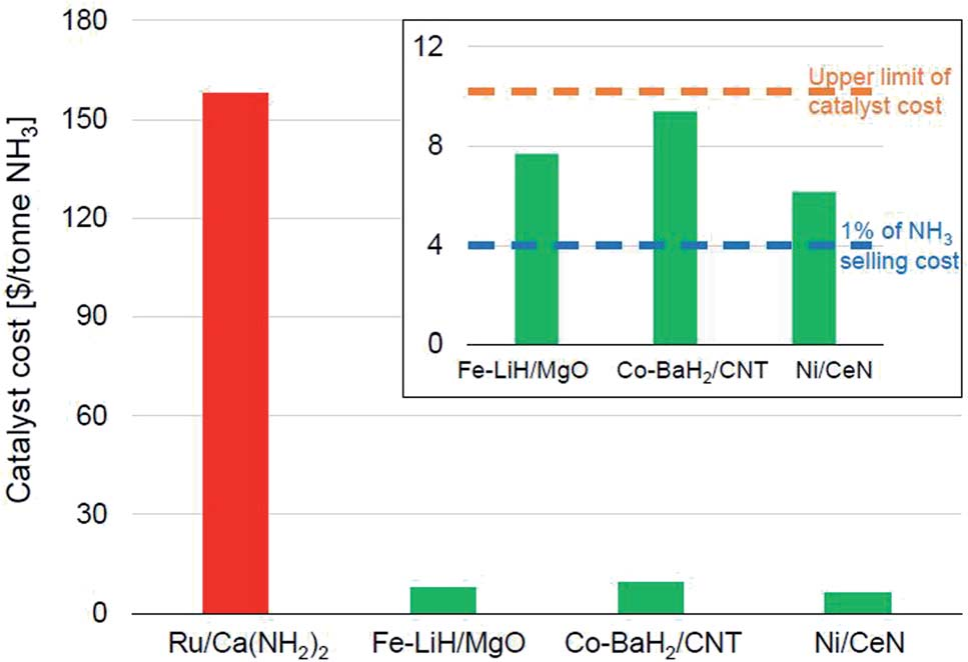
Catalyst costs per tonne of ammonia produced assuming a catalyst lifetime of 6 months. The catalyst activity data was taken from the following references: Ru/Ca(NH_2_)_2_,^70^ Fe–LiH/MgO,^112^ Co–BaH_2_/CNT,^99^ and Ni/CeN.^86^ The cost of the materials per kg were taken as $400, $8, $15 and $10 respectively, which are assumed from current element costs^125^ and previously reported costs of similar materials.^17^

Fig. 6 shows the catalyst costs incurred per tonne of ammonia for some of the best-performing newer-generation catalysts assuming a material lifetime of 6 months. We would like to emphasize that this lifetime is more of an arbitrary assumption generated from the catalyst consumption analysis presented in the preceding paragraph and must not be viewed as a verdict on the expected stability of these materials. We had noted that Ru-based catalysts were the most efficient for lower temperature ammonia synthesis (Fig. 4), also resulting in the highest productivities (Fig. 5). This impressive performance notwithstanding, the high cost of ruthenium poses serious challenges for these materials to be industrially relevant. This also potentially explains the lower penetration of the KAAP catalyst despite higher activity in HB synthesis. A 10 wt% Ru/Ca(NH_2_)_2_ catalyst with a productivity of 34 000 μmol NH_3_ per g_cat_ per h and a lifetime of 6 months overshoots the upper limit on catalyst cost ($10 per tonne-NH_3_) by more than an order of magnitude (Fig. 6). In the framework of the assumptions underpinning the catalyst costs derived in Fig. 6, the ruthenium-based catalyst would need to have a lifetime of close to a decade for its cost to fall below $10 per tonne-NH_3_. Therefore, for these catalysts to be of industrial interest, it is paramount that alongside any further improvements in catalyst productivity, significant attention must be directed towards their long-term stability. In contrast, other TM-based catalysts, despite being less efficient, compare more favorably in terms of their economic potential. The Fe-, Co- and Ni-based catalysts depicted in Fig. 6 cost less than $10 per tonne-NH_3_; however, for an ammonia selling price of $400 per tonne, these catalysts still represent over 1% of the final selling cost, which as we elaborate below, is still a significant expenditure in the economics of ammonia manufacture.

In the economics of ‘green ammonia’ manufacture, the cost contribution of the synthesis loop *i.e.* the catalytic conversion of hydrogen and nitrogen to ammonia is typically a very small fraction of the total manufacturing cost. On-site hydrogen production using renewable energy sources is the most significant contributor to total operating as well as capital costs, with the latter depending on the origin of water and the level of pre-treatment required before electrolysis.^17,126,127^ For example, in a simulation of a small-scale HB plant, the capital cost for hydrogen production constitutes nearly 60% of the total capital cost for ammonia manufacture. In comparison, that of the ammonia synthesis reactor is a mere 2%.^126^ Likewise, over 90% of the total energy consumed for ammonia manufacture is exclusively for hydrogen production.^126^ These numbers identify the bottleneck in the economically efficient production of green ammonia to be renewable hydrogen production and not the ammonia synthesis loop. As a result, the allowance for ammonia synthesis catalyst costs is expected to be even more stringent than in other industrial catalytic processes. In the simulation of the small-scale HB process cited above, the cost of the iron catalyst amounts to only 0.3% of the total annual capital cost of the ammonia synthesis process, which is estimated based on the installed costs of equipment and the cost of the catalyst. Despite that, the ammonia produced through this pathway is twice as expensive as conventional commodity ammonia.^126^ While the greater levelized cost of green ammonia has less to do with the ammonia synthesis loop *per se*, this shows that catalysts costing over 1% of the ammonia selling price could significantly hamper the economic viability of the process. Therefore, while the TM-based catalysts shown in Fig. 6 cost less than $10 per tonne-NH_3_, when a lifetime of 6 months is assumed, these costs are still appreciable and efforts to decrease them further should be made.

In terms of the catalyst consumption criterion introduced earlier, this calls for targeting a much lower value than the upper limit, especially with newer catalyst formulations expected to cost more than conventional fused iron catalysts. For instance, a catalyst that costs $40 per kg and is consumed at the rate of 0.05 kg_cat_ per tonne_product_ results in a promising catalyst cost of $2 per tonne-NH_3_. However, assuming a productivity of 34 000 μmol NH_3_ per g_cat_ per h, such a catalyst consumption can only be realized if the catalyst is stable for nearly 4 years on stream. Despite being rudimentary in nature, the economic analysis presented herein unequivocally underscores the importance to take on board material lifetime considerations in the quest for improved catalyst formulations for ammonia synthesis. Unlike classical iron-based HB catalysts, which generally have a long service life, and whose low cost make any investigations into regenerating deactivated catalysts redundant, the higher price (and related material scarcity) of some of the more recent and sophisticated catalyst materials could potentially establish a greater scope for catalyst deactivation–regeneration processes. In the eventuality of the newer-generation catalysts being unable to match HB catalysts for their time-on-stream performance, an industrially feasible pathway to reverse deactivation could lower catalyst consumption and keep such materials in contention for industrial application. Importantly, catalyst deactivation mechanisms in green ammonia manufacture are expected to vary from those prevailing in conventional ammonia synthesis, since the impurities associated with hydrocarbon-derived hydrogen are usually not present in hydrogen manufactured by water electrolysis. While the higher purity of hydrogen generated by the latter route can be considered as an advantage to combat catalyst deactivation, it is worth noting that the performance of the more novel catalysts as reported in published literature is studied under very pure hydrogen and nitrogen, typically >99.9999%.

A rigorous techno-economic assessment, which is beyond the scope of this perspective, should be able to define more precise catalyst cost and consumption targets as well as the scope for catalyst regeneration practices. Any such assessment must meticulously account for the disparity in catalyst costs and performance at lower temperatures and pressures. The importance of doing so is evident from a sensitivity analysis performed by Bañares-Alcántara *et al.*, where the variation in the cost of the ammonia synthesis loop for three different catalyst options (Fe, Ru and Co_3_Mo_3_N) were studied.^17^ The higher activity of Co_3_Mo_3_N when compared to iron under milder conditions will allow lowering the operating pressure, which cuts the costs incurred by compressors in the ammonia synthesis loop by half. Albeit being relatively more active than iron under these conditions, the catalytic performance of Co_3_Mo_3_N is far from satisfactory. The lower single-pass conversion under milder conditions implies a larger reactor and recycle loop, considerably increasing the expenditure made towards the reactor and flash vessels as well as the Co_3_Mo_3_N catalyst. As a result of this and the higher material cost of Co_3_Mo_3_N compared to iron, the net expenditure on the ammonia synthesis loop employing a Co_3_Mo_3_N catalyst under milder conditions surpasses that using iron at higher pressures.^17^ This underscores the need to develop cheaper and more active catalysts that can help accomplish a higher single-pass conversion approaching the equilibrium upper limit under milder conditions. This also calls for a diligent analysis of the interplay of capital expenditure and catalyst performance in determining the cost competitiveness of the process. The derivation of performance benchmarks for ammonia synthesis catalysts that is based on such an analysis will be able to identify industrially promising windows of operation; for instance, if certain pressure ranges are more favorable than others. While decreasing the operating pressure will cut compressor costs, the lower partial pressure of ammonia at the reactor outlet could require extra refrigeration energy (assuming product separation is achieved by condensation). The catalyst must enable a conversion where the compressor energy savings offset the extra energy requirements required downstream. Therefore, catalyst performance benchmarks derived on the basis of an integrated approach considering the impacts to the overall synthesis loop are expected to be more useful.

## Conclusions and future outlook

The Haber–Bosch technology for ammonia synthesis has seen only relatively modest changes from a catalysis perspective in over a century of operation. However, the urgent need to eliminate the large carbon footprint of ammonia production is prompting widespread consideration of possible improvements to the process, including smaller scale production. In addition to decarbonizing fertilizer production, green ammonia production can also facilitate the use of ammonia as a fuel and a hydrogen store, given its mature, simple and widespread distribution infrastructure. While more dramatic technological changes such as electrochemical ammonia synthesis are under development, we have considered the potential of advanced heterogeneous catalysts to contribute to this transformation.

The decarbonisation of thermal ammonia production hinges on a transition to hydrogen production *via* water electrolysis, which in turn has important implications for catalytic ammonia synthesis. The modular nature of water electrolysis units and the intermittency associated with renewable energy sources make this route of hydrogen production more suited to operation on a small scale. In contrast, the severe reaction conditions that characterize HB ammonia synthesis render it more appropriate for large-scale, steady-state operation. An approach to synthesize ammonia at lower temperatures and pressures will not only help alleviate this issue of scale mismatch but also lower the energy consumption of the overall process, which despite significant improvements over the years, remains fairly high.

As we illustrate in our discussion, conventional transition metal catalysts perform poorly under these milder conditions, prompting extensive research into ways to improve catalyst activity. The mediocre performance is attributed to the sluggish kinetics of nitrogen dissociation (or activation) under these conditions. A more nuanced description of this limitation arises from the linear scaling relationship that exists between nitrogen adsorption energy and the transition-state energy (strong activation = slow desorption). As briefly summarized in this perspective, multiple approaches to expediting nitrogen dissociation have been reported, which can broadly be classified as attempts to either ‘shift’ or ‘break’ the scaling relation governing nitrogen activation. The former typically involves the use of a promoter, co-catalyst or support that increases the electron density on the transition metal, thereby facilitating the cleavage of the nitrogen–nitrogen triple bond. In the case of the latter, there is usually a spatial separation of nitrogen activation and hydrogen activation, which enables the hydrogenation of the adsorbed nitrogen species to take place at a different site from the one which activates nitrogen.

The performance of newer-generation catalysts adopting such strategies has often been juxtaposed with the more classical iron- and ruthenium-based catalysts in literature to demonstrate the considerable improvements that have been made. In this perspective, we went beyond such a relative comparison of productivities to evaluate the performance of catalysts in terms of their efficiency. This revealed several highly efficient catalysts at a temperature of 673 K and a pressure no greater than 10 bar. However, below 613 K, most catalysts report mediocre efficiencies. The handful of catalysts that buck this trend tend to address the issue of hydrogen activation (equivalently hydrogen adsorption–desorption) in conjunction with that of nitrogen activation. This more comprehensive approach to catalyst design, attaining fractional reaction orders in both nitrogen and hydrogen, along with a reasonably low overall activation energy for ammonia synthesis, shows significant promise. We also emphasized the importance of several physical factors in achieving a high catalyst efficiency, including that of specific surface area and porosity. While microscopic considerations of hydrogen and nitrogen activation help enhance the intrinsic catalytic activity (site TOF), maximizing the accessibility and concentration of active sites in a material is vital to achieving high efficiency.

An efficient catalyst is indispensable to realize economically feasible green ammonia manufacture, but is not the only determining factor. By comparing the performance of state-of-the-art ammonia synthesis catalysts with pre-defined industrial viability targets for catalytic processes, we showed that the best productivities attained to date lie more towards the lower bound of the catalyst activity criterion. This has ramifications for the catalyst consumption targets, because unless these materials have a relatively long lifetime – in the order of years – the costs incurred by the catalysts can preclude the viability of the process. This is particularly important for ruthenium-based catalysts that tend to be much more expensive than other TMs. Nevertheless, even the cheaper iron- and cobalt-based catalysts need to have a lifetime of at least a year to ensure that catalyst costs fall well below $4 per tonne-NH_3_. The analysis presented herein unambiguously conveys the importance to marry long lifetime with high activity for catalyst development in the future. Unless both these considerations are taken into account, it might be difficult for many of the more novel catalysts to rise beyond academic interest. This is particularly relevant for the more sophisticated catalyst formulations with multiple or variable active sites identified in the recent past, wherein ensuring sustained intimate contact between the catalyst and co-catalyst phases is often a challenge. With academic labs typically not being equipped to run long-term stability tests, seeking assistance from national testing facilities could help bridge the gap between academia and industry and accelerate the adoption of next-generation catalyst materials.

Going forward, we recommend formulating more precise quantitative targets for catalyst cost and consumption in the context of ammonia manufacture. The varying degrees of complexity in the newer catalyst formulations, the widely differing costs of transition metals, and the necessity for the ammonia synthesis loop to constitute only a small fraction of the total manufacturing cost warrant such an analysis, which can prescribe goalposts that next-generation catalysts must strive to reach. This would establish a better yardstick to evaluate the potential of these catalyst systems for industrial green ammonia manufacture, as opposed to the widely prevalent practice of a simplistic comparison of the activity of the newer materials to classical HB catalysts. To conclude, the next few years of research are key to developing improved heterogeneous catalysts for green ammonia manufacture in the global context of net-zero targets by 2050. On the basis of the analysis presented in this perspective, we believe the following considerations will shape upcoming catalysis research in this discipline: (i) holistically address the issues of nitrogen activation, hydrogen activation, and hydrogenation of adsorbed nitrogen species to tailor active sites with high intrinsic activity, (ii) smart synthesis and engineering of materials that maximizes the utilization of such active sites, and (iii) improve the long-term stability of catalysts—particularly in the context of new impurity profiles with green hydrogen supplies and potential ramped operation when coupled to renewables at smaller scales—to ensure a low catalyst consumption and render them cost-competitive.

## Author contributions

Conceptualization and investigation: MR and JWM. Writing – original draft: MR. Writing – review & editing – MR and JWM.

## Conflicts of interest

There are no conflicts to declare.

## Supplementary Material
